# Broad-spectrum bioactivities and therapeutic potential of essential oils of certain aromatic and medicinal plants and their combination

**DOI:** 10.1038/s41598-025-18314-1

**Published:** 2025-09-12

**Authors:** Mohga S. Abdalla, Aliaa Abdelrafea Salem, Eman Tawfik Hussien, Shimaa S. Ramadan

**Affiliations:** 1 Chemistry Department, Faculty of Science, Helwan University, Cairo, Egypt; 2 Botany and Microbiology Department, Faculty of Science, Helwan University, Cairo, Egypt

**Keywords:** *Mentha canadensis*, *Corymbia citriodora*, *Plectranthus amboinicus*, Antiviral, Anticancer, Antidiabetic, Antioxidant, GC–MS, Essential oil, Biochemistry, Biotechnology, Drug discovery, Plant sciences

## Abstract

This research investigates the bioactive properties of essential oils obtained from three medicinal and aromatic species: *Mentha canadensis*, *Corymbia citriodora*, and *Plectranthus amboinicus*. The essential oils were evaluated for antiviral, antioxidant, anticancer, and antidiabetic activities. The gas chromatography-mass spectrometry (GC–MS) examination identified a variety of phytochemical constituents, including thymol, citronellol, and levomenthol. Cytopathic impact studies indicated that *M. canadensis* and *C. citriodora* oils attained a maximum of 35.34% suppression of Adeno 7 virus replication. The oils exhibited minimal cytotoxicity in Vero cells, with viability exceeding 97% at concentrations of ≤ 312 µg/mL. *P. amboinicus* oil had the highest cytotoxicity against H1299 lung cancer cells (IC_50_ = 11 µg/mL), indicating significant anticancer efficacy. The antidiabetic effects were evidenced by α-glucosidase inhibition, with *P. amboinicus* oil displaying the greatest activity (IC_50_ = 248.1 µg/mL). The DPPH experiment demonstrated that *P. amboinicus* exhibited the highest antioxidant activity (IC_50_ = 5923 µg/mL). The results highlight the medicinal potential of these essential oils, including *P. amboinicus* for anticancer, antioxidant and antidiabetic applications, and *C. citriodora* and *M. canadensis*for antiviral purposes. The current work serves about four goals of the sustainable development goals (SDG) as follow: SDG 3: Good Health and Well-being, SDG 12: Responsible Consumption and Production, SDG 9: Industry, Innovation, and Infrastructure and SDG 15: Life on Land.

## Introduction

The plant-based traditional medicine system remains integral to healthcare, with over 80% of the global population primarily depending on traditional medicines or primary health care. Medicinal plants are flora that possess intrinsic active compounds utilized for illness treatment or pain alleviation. The utilization of traditional medicines and medicinal plants as therapeutic agents for health maintenance is prevalent in many underdeveloped nations. The modern pharmacopoeia comprises at least 25% of medications sourced from plants, with many others being synthetic equivalents based on prototype chemicals extracted from plants^1,2^. The therapeutic qualities of plants may be attributed to the antioxidant, antibacterial, and antipyretic activities of their phytochemicals. The World Health Organization asserts that medicinal plants may serve as the optimal source for a diverse array of pharmaceuticals. Consequently, these plants warrant investigation to enhance comprehension of their features, safety, and efficacy^3,4^.

Medicinal plants generate bioactive chemicals mostly utilized for therapeutic applications. These substances either affect many systems of animals, including humans, or interfere with the metabolism of the bacteria infecting them. The bacteria might be either harmful or symbiotic. In each case, it regulates host-microbe interactions to benefit the host. The discovery, isolation, purification, and characterization of bioactive chemicals in plants by diverse analytical methods is essential^5^.

The limitations of traditional medical management of diseases highlight the clear need for safe and effective remedies. Herbal medications and their derived natural compounds may offer future therapies for numerous ailments^6^.

The *Mentha* species examined in this study have been traditionally utilized for the treatment of many ailments, including gastrointestinal disorders, respiratory conditions, infectious diseases, inflammatory disorders, and menstrual issues across diverse cultures globally^7^. Essential oils play a crucial role in nature by safeguarding plants through their antifungal, antibacterial, antiviral, and insecticidal activities, as well as by deterring herbivores by diminishing their hunger for plants exhibiting these characteristics. Health and Human Services Public Health Services have acknowledged essential oils as safe substances, and certain essential oils possess constituents that can function as antibacterial agents. Several studies have shown that essential oils (EOs) can inhibit viruses and food pollutants, which suggests that they could be useful in the food sector^8^.

Essential oils (EOs) have antibacterial, antioxidant, and antiproliferative properties, which may be utilized, either independently or in conjunction with other agents, for the formulation of novel pharmaceuticals. Nonetheless, their chemical heterogeneity, influenced by species, variations, or geographical origin, among other considerations, dictates distinct bioactivities that require assessment^9,10^. Essential oils, containing components with established antibacterial properties such as menthone, piperitone oxide, carvone, and linalool, are extensively documented for their significant antimicrobial efficacy. Menthone, piperitenone oxide, and carvone exhibited significant antibacterial properties. Pulegone is a monoterpene ketone found in the essential oils of various mint species. Pulegone is the second principal ingredient of the essential oil derived from our material. Plugone has been utilized as a flavoring additive in food and beverages, as well as an ingredient in fragrance goods and flea repellents^11,12^.

In order to investigate the potential of essential oils from *Mentha canadensis*, *Corymbia citriodora*, and *Plectranthus amboinicus* as natural therapeutic agents for potential pharmaceutical uses, this study compared and evaluated their bioactive qualities, with a particular emphasis on their antiviral, antioxidant, antidiabetic, and anticancer activities. The three plant species—*Mentha canadensis*, *Corymbia citriodora*, and *Plectranthus amboinicus*—were chosen because they are ethnobotanically important, easy to find, and have well-known bioactive qualities. In the area where the study took place, all three species are easy to find and have been used in traditional medicine for a long time to fight infections, reduce inflammation, and protect against free radicals. They are also aromatic and medicinal plants that are high in essential oils and phenolic compounds, which are important for the goals of this study. It is easy to grow and care for them in the local climate, which makes them good and long-lasting experimental models.

## Materials and methods

### Plant materials

This study focused on three medicinal and ornamental plants: *Mentha canadensis*, *Corymbia citriodora*, and *Plectranthus amboinicus*. The plants were brought from “VitroLab” for plant tissue culture purposes, located on Masr-Alex desert road, Egypt. The plants were introduced at four weeks of age on MS medium. The MS media used in this study is ready media, commercially purchased from “Caisson” – USA, with lot number: 09240005.

### Extraction of essential oils

Three essential oils were extracted via hydro-distillation of 50 g of fresh plant materials of *Mentha canadensis*, *Corymbia citriodora*, and *Plectranthus amboinicus*, separately (including leaves and stems) for 1.5 h, employing Clevenger equipment. The extracted oils were kept at 4 °C for preservation until further analysis.

The essential oil combination was applied according to the synergistic chemical compositions and documented bioactivities of the component oils. *Plectranthus amboinicus* is abundant in thymol and esters exhibiting significant antimicrobial and antioxidant properties; *Corymbia citriodora* possesses elevated concentrations of citronellal and associated aldehydes with powerful antimicrobial effects; and *Mentha canadensis* serves as a source of menthol and cyclohexanone derivatives recognized for their membrane-disruptive and anti-inflammatory activities. The amalgamation of these oils aimed to investigate possible synergistic effects from several kinds of bioactive chemicals, utilizing non-toxic amounts determined in initial cytotoxicity studies.

### Gas chromatography–mass spectrometry analysis

Mass spectrometry analysis was performed with a Shimadzu GCMS-QP2010 system (Kyoto, Japan), fitted with a Rtx-5MS fused silica capillary column (30 m × 0.25 mm internal diameter × 0.25 µm film thickness; Restek, USA) and a split–splitless injector. The column temperature was initially sustained at 45 °C for 2 min under isothermal conditions, thereafter, increasing to 300 °C at a rate of 5 °C per minute. The temperature was subsequently maintained at 300 °C for an additional 5 min under isothermal conditions. The injector temperature was established at 250 °C, and the flow rate of the helium carrier gas was sustained at 1.41 mL/min. Mass spectra were obtained using the following operational parameters: filament emission current of 60 mA, ionization voltage of 70 eV, and ion source temperature of 200 °C. Samples were diluted to a 1% (v/v) concentration and injected in split mode with a 1:15 split ratio.

### Metabolic activities

Some essential metabolic activities were evaluated from the ethanol extract of the three plant species. These metabolic activities included antimicrobial, antiviral, antioxidant, antidiabetic and anticancer activities.

### Antiviral activity

#### Cytotoxicity

The cytotoxic activity of the essential oils (EOs) was assessed using the MTT assay, with the EOs applied at a concentration of 200 mg/ml. MTT stands for “3-(4,5-dimethylthiazol-2-yl)-2,5-diphenyltetrazolium bromide assay.” It’s a colorimetric test that is often used to check how well cells are alive and growing, as well as how medications affect cells in a harmful way. EOs were two-fold serially diluted and applied to pre-cultured Vero cell lines for a 24-h incubation period at 37 °C after the removal of the growth medium. Post-treatment, the Vero cell lines were examined microscopically to detect any morphological alterations or the presence of detached cells. Dead cells were rinsed off by washing with phosphate-buffered saline (PBS) at pH 7.2 ± 0.2, supplemented with 0.05% Tween. The residual viable cells were treated with 0.5% MTT stain, at 25 µL per well. Plates were then incubated for 3–4 h at 37 °C. The developed intra-cytoplasmic MTT formazan crystals were solubilized using 0.05 mL of dimethyl sulfoxide (DMSO) for 30 min on a plate shaker. The optical densities were measured using an ELISA plate reader (Biotek – 800, USA). The IC50 of test extracts was determined using the Master–plex-2010 program (ver. 2.0.0.77). Data was collected from three independent experiments, as described by Berridge et al.^13^. The percentage of cell viability was calculated using the following formula:

$${\text{Cell}}\;{\text{viability }}\left( {{\% }} \right){ } = { }\left( {\frac{{Optical\;Density\;\left( {OD} \right)\;of\;Treated\;Cells}}{{Optical\;Density\;\left( {OD} \right)\;of\;Untreated\;Cells}}} \right) \times 100$$

The cytotoxic concentration (CC50) of the EOs was also determined using the Master–plex-2010 software (ver. 2.0.0.77)^14^.

### Evaluation of antiviral activity of the essential oils against adeno 7 virus

The antiviral activity of the essential oils (EOs) against Adeno 7 virus was evaluated according to Petricevich and Mendonça^15^. The assessment involved titrating the virus in both the presence and absence of the Eos. The difference in viral titters indicates the antiviral efficacy of the Eos. Vero cells were pre-treated with the EOs for 24 h prior to viral inoculation to determine the impact of the EOs on the early stages of viral replication, as follows: Vero cells were counted at a density of 10^5^ cells/mL and cultured in 96-well plates. Upon reaching confluency (3.66 µg/ml with percentage of 35.34%), the growth media were discarded, and the cells were treated with non-toxic concentrations of the EOs (100 µL/well) for 24 h at 37 °C. Meanwhile, control plates remained untreated for viral control titration.

The Adeno 7 virus was serially diluted tenfold in E-MEM. The growth media was removed, and each viral dilution was injected at 0.1ml/well in Vero cell cultures, both in the presence and absence of the EOs. Untreated and uninfected wells were included as negative controls. The plates were incubated at 37 °C and monitored daily using an inverted microscope. After three days, the viral titters in both treated and untreated cells were evaluated using the cytopathic effect (CPE) endpoint assay, as described by Steven et al.^16^. The virus infectivity assay, based on the CPE endpoint method, involved identifying the highest virus dilution that induced CPE in 50% of cell cultures. This endpoint, known as the 50% cell culture infectious dose (CCID₅₀), was calculated using the Reed and Muench^17^ formula:

$${\text{CCID}}50 = \left( {\frac{{{\text{Percentage}}\;{\text{of}}\;{\text{CPE}} > 50{{\% }} - 50}}{{{\text{Percentage}}\;{\text{of}}\;{\text{CPE}} > 50{{\% }} - {\text{Percentage}}\;{\text{of}}\;{\text{CPE}} < 50{{\% }}}}} \right) \times {\text{log}}\;{\text{dilution}}$$

All titrations were performed in triplicate. The discrepancies between the mean virus titers in treated and untreated plates indicate antiviral action.

### Anticancer activity

#### Cell culture

The essential oils were evaluated for their activity against H1299 lung cancer cells. The cell culture was maintained in Dulbecco’s Modified Eagle Medium (DMEM) supplemented with streptomycin, penicillin, and 10% fetal bovine serum. The cells were incubated under a humidified atmosphere of 5% (v/v) CO2 in air at a constant temperature of 37 °C.

#### Cell viability assay

Cells were plated in 96-well plates at a density of 5 × 10^3^ cells per well and allowed to adhere by incubating in culture media for 24 h. Following this, the cells were treated with essential oils (EOs) at varying concentrations ranging from 0 to 60 μg/ml and incubated for an additional 72 h. After the treatment period, the media was replaced with 10% trichloroacetic acid (TCA) and incubated for 1 h to fix the cells. The TCA was subsequently removed, and the cells were washed thoroughly with distilled water. Next, 70 μl of sulforhodamine B (SRB) solution (0.4% w/v) was added to each well and allowed to stain the cells for 10 min. The plates were then washed with 1% acetic acid to remove unbound dye and left to air-dry overnight. Finally, 10 mM tris(hydroxymethyl)aminomethane (TRIS) buffer was added to solubilize the bound dye, and the absorbance was measured using a BMG LABTECH- FLUOstar Omega microplate reader (https://www.bmglabtech.com/en/fluostar-omega/) (Ortenberg, Germany).

### Antioxidant activity

The essential oils were initially prepared at a concentration of 50 mg/mL in methanol. Sample 1 was utilized at a final concentration of 25 mg/mL, while Samples 2, 3, and 4 were serially diluted in methanol to achieve final concentrations of 25,000, 12,500, 6250, 3125, and 1562.5 μg/mL. A stock solution of Trolox, with a concentration of 40 µg/mL, was prepared in methanol, from which five working concentrations were derived: 12.5, 6.25, 3.125, 1.5625, and 0.78125 µg/mL.

The DPPH (2,2-diphenyl-1-picryl-hydrazyl-hydrate) free radical assay was conducted following the methodology outlined by Boly et al.^18^, with modifications as described by Elkholy et al.^19^. Briefly, 100 µL of freshly prepared DPPH reagent (0.1% in methanol) was added to 100 µL of each sample in a 96-well plate (n = 3). The reaction mixture was incubated at room temperature for 30 min in the dark. After incubation, the reduction in DPPH color intensity was measured spectrophotometrically at 540 nm. The percentage inhibition of DPPH radicals was calculated using the following equation:

$${\text{Percentage}}\;{\text{inhibition}} = \left( {\frac{{{\text{Average}}\;{\text{absorbance}}\;{\text{of}}\;{\text{blank}} - {\text{Average}}\;{\text{absorbance}}\;{\text{of}}\;{\text{the}}\;{\text{test}}}}{{{\text{Average}}\;{\text{absorbance}}\;{\text{of}}\;{\text{blank}}}}} \right) \times 100$$

Data were expressed as means ± standard deviation (SD). Absorbance measurements were recorded using a FluoStar Omega microplate reader.

### Antidiabetic activity

#### Standards and samples preparation

A stock solution of acarbose was prepared at a concentration of 1000 µg/mL in phosphate buffer (100 mM, pH 7.0). From this stock solution, final concentrations of 31.25, 62.5, 125, 250, and 500 µg/mL were prepared by dilution in water. Samples 2, 3, and 4 were dissolved in methanol to achieve final concentrations of 1000, 500, 250, 125, and 100 µg/mL. Similarly, Sample 1 was dissolved in methanol to yield final concentrations of 1000 and 100 µg/mL.

#### α-Glucosidase inhibitor assay

The assay was conducted following the methodology described by Abdallah et al.^20^. Briefly, in 96-well microplates, 25 μL of the samples or blank were incubated with 50 μL of α-glucosidase enzyme (0.6 U/mL, sourced from *Saccharomyces cerevisiae*) in phosphate buffer (0.1 M, pH 7.0) for 10 min at 37 °C. Subsequently, 25 μL of 3 mM para-nitrophenyl β-D-glucopyranoside (pNPG) dissolved in phosphate buffer (pH 7.0) was added as a substrate, and the mixture was further incubated for 5 min at 37 °C. The enzymatic activity was quantified by measuring the release of p-nitrophenol from the pNPG substrate at an absorbance wavelength of 405 nm using a microplate reader (Omega, USA).

The percentage inhibition of α-glucosidase activity was calculated using the following formula:

$$\% {\text{Inhibition}} = \left( {\frac{{{\text{A}}\;{\text{blank}} - {\text{A}}\;{\text{sample}}}}{{{\text{A }}\;{\text{blank}}}}} \right) \times 100$$

Where A blank represents the absorbance of the control (blank, without inhibitor) and A sample denotes the absorbance in the presence of the inhibitor.

### Statistical analysis

For all experiments, results were represented as the mean ± standard deviation of three independent experiments. Statistical significance was determined using One Way ANOVA. Differences at P values less than 0.05 were considered significant using Minitab (19).

## Results

### Plant materials

**Fig. 1:**
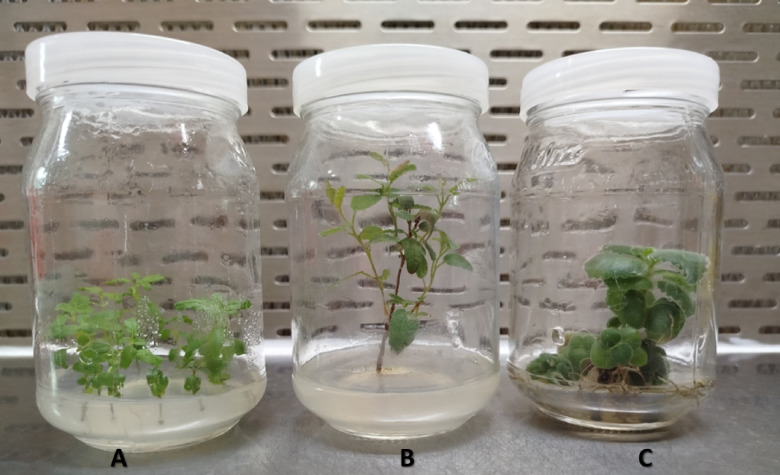
In vitro culture of three fragrant and medicinal plants (**a**. *Mentha canadensis*, **b**. *Corymbia citriodora*, and **c**. *Plectranthus amboinicus*).

Figure 1 showed the in vitro culture of three aromatic and medicinal plant species utilized in this study: (a) *Mentha canadensis*, (b) *Corymbia citriodora*, and (c) *Plectranthus amboinicus*. The plants plant cultured on ¾ MS media supplemented with 0.5 ml/L BAP and 1ml/L Kin at the age of 4 weeks. Each species was successfully cultivated under sterile conditions, demonstrating robust growth and distinct physical characteristics. These cultures were used as the starting point for later experiments. The plantlets showed well differentiation of plant structure of shooting and rooting.

### Gas chromatography-mass spectrometry (GC–MS) analysis

**Fig. 2:**
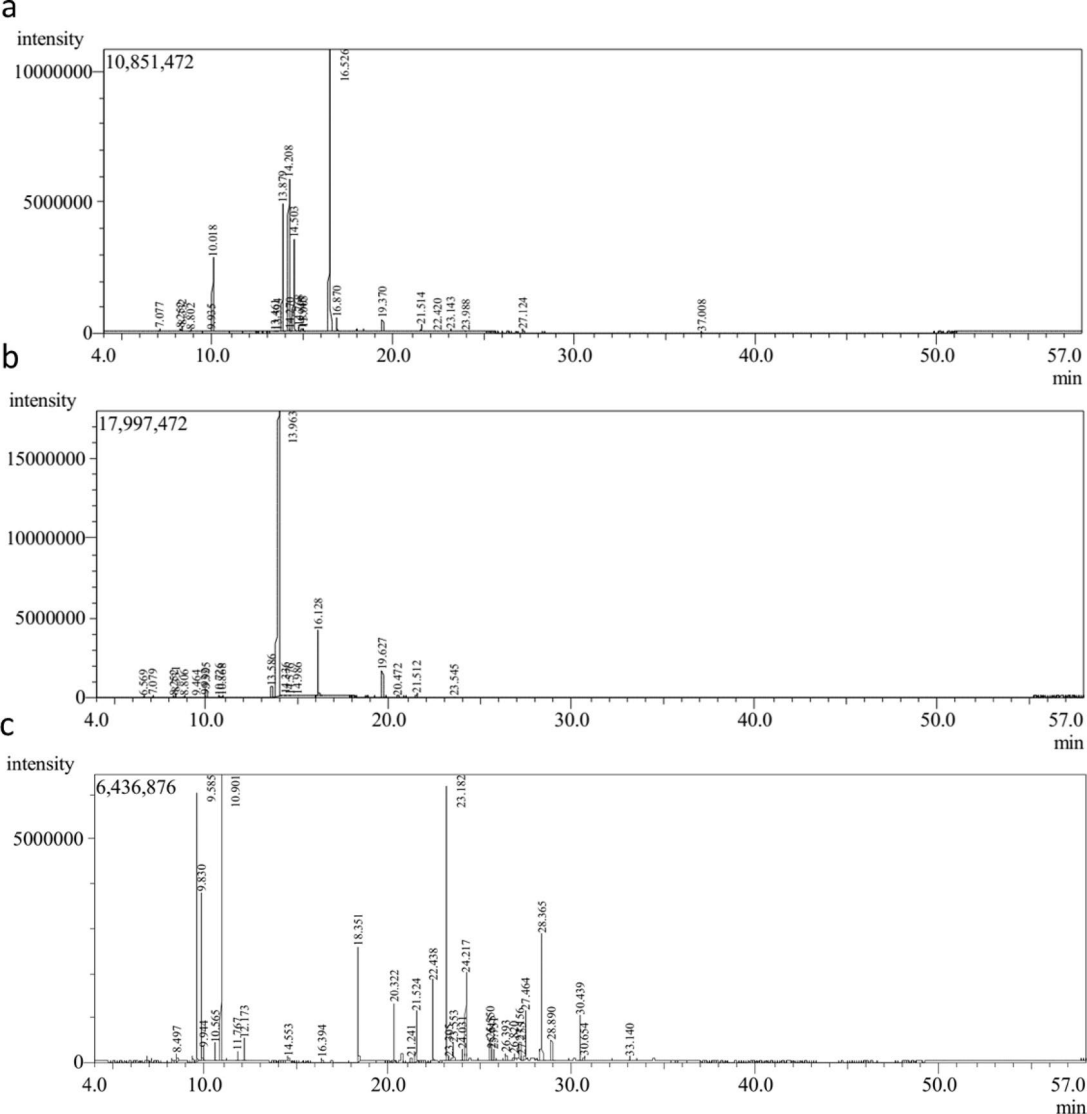
Gas chromatography-mass spectrometry (GC–MS) chromatograms of compounds present in essential oils of (**a**) *M. canadensis*, (**b**) *C. citriodora* and (**c**) *P. amboinicus*.

The chemical compositions of the essential oils were determined by GC–MS analysis; *M. canadensis* oil contained 10.21% levomenthol and 13.24–16.66% cyclohexanone derivatives. *P. amboinicus* oil had 6.39% thymol, but C. citriodora oil contained 84.06% 6-octenal and 9.0% citronellol. Cyclohexanol and 5-methyl-2-(1-methylethyl)- were among the chemicals that showed antibacterial activity; they most likely interacted with bacterial membranes. In *P. amboinicus*, propanoic acid, 2-methyl-butyl ester, 1,6-Octadien-3-ol, and 3,7-dimethyl are expected to interact with membranes or be enzyme inhibitors (Fig. 2, Table 1).

**Table 1:** Bioactive constituents of *M. canadensis*, *C. citriodora* and *P. amboinicus* essential oils identified by GC–MS analysis.

Plant	Retension time	Area %	Compounds	m/z
*M. canadensis*	7.077	0.24	Alpha-Pinene	93.1
	8.252	0.22	Bicyc10[3.1.0]hexane, 4-methylene-l -(1 -methylethyl)-	93.1
	8.332	0.44	Beta-Pinene	93.1
	8.802	0.15	Beta.-Myrcene	41.05
	9.935	0.12	Limonene	68.05
	10.018	5.36	Eucalyptol	43
	13.461	0.05	Bicyclo[3.1.0]hexan-3–01, 4-methylene-l-(l -methylethyl)-, [ IS-(I .alpha.,3.beta.,5.alph	91.1
	13.594	0.14	Cyclohexanol, 2-methyl-5-(l -methylethenyl)-, (l.alpha.,2.beta.,5 .alpha.)-	41
	13.879	13.24	Cyclohexanone, 5-methyl-2-( 1 -methylethyl)-	41.05
	14.208	16.66	Cyclohexanone, 5-methyl-2-( 1 -methylethyl)-	41.05
	14.27	0.15	Cyclohexanemethanol, .alpha.,.alpha.-d1methyl-4-methylene-	59.05
	14.503	10.21	Levomenthol	41.05
	14.768	0.37	Cyclohexanol, 5-methyl-2-(l -methylethyl)-, [IS-( l.alpha.,2.alplL,5.beta.)]-	41.05
	15.003	0.35	Alpha.-Terpineol	59.1
	16.526	48.78	Cyclohexanone, 5-methyl-2-( 1 -methylethylidene)-	81.1
	16.87	1.07	2-Cyclohexen-l -one, 3-methyl-6-(l -methylethyl)-	82.05
	19.37	1.08	2-Cyclohexen-l -one, 3-methyl-6-(l -methylethylidene)-	107.1
	21.514	0.56	Caryophyllene	41.05
	22.42	0.03	Humulene	93.15
	23.143	0.23	1,6-Cyclodecadiene, 1 -methyl-5-methylene-8-(l -methylethyl)-, [S-(E,E)]-	91.1
	23.988	0.06	Naphthalene, 1 ,2,4a,5,6,8a-hexahydro-4,7-dimethyl-l-(l-methylethyl)-	161.2
	27.124	0.25	Beta-copaene	161.2
	37.008	0.04	Isoaromadendrene epoxide	41.05
*C. citriodora*	6.569	0.04	Propanoic acid, 2-methyl-, butyl ester	43.05
	7.079	0.09	Alpha Pmene	93.1
	8.252	0.05	Beta-Pinene	93.1
	8.331	0.35	Beta-Pinene	93.1
	8.806	0.08	Beta-Myrcene	41.05
	9.932	0.02	Limonene	68.1
	9.995	0.12	Eucalyptol	43
	10.726	0.78	3-Hexen-l -01	41.05
	10.868	0.08	Gamma-Terpinene	93.1
	13.586	0.05	Isopulegol	41.05
	13.963	1.44	6-Octenal, 3,7-dimethyl-, (R)-	41.05
	14.336	84.06	Isopulegol	41.05
	14.576	0.16	5-1sopropyl-2-methylbicyc10[3. 1 .0]hexan-2–01 #	71.1
	14.986	0.1	Alpha-Terpineol	59.05
	16.128	0.16	Citronellol	41.05
	19.627	9	6-Octen-l -01,3,7-dimethyl-, acetate	43.05
	20.472	2.93	Geranyl isovalerate	41.05
	21.512	0.1	Caryophyllene	41.05
	23.545	0.37	Elemene	121.15
*P. amboinicus*	8.497	0.39	1-Octen-3-01	57.05
	9.585	12.12	Cyclohexene, 1-methy14-(l-methylethylidene)-	93.1
	9.83	7.47	p-Cymene	119.15
	9.944	0.66	Alpha-Pinene	93.1
	10.565	0.91	Beta-Ocimene	93.1
	10.901	13.97	Gamma-Terpinene	93.1
	11.767	0.53	Bicyclo[2.2.1]heptan-2-0ne, 1,3,3-trimethyl-	81.1
	12.173	1.15	1 ,6-Octadien-3-01, 3,7-dimethyl-	41.05
	14.553	0.28	Terpinen4-01	71.05
	16.394	0.22	Alpha-frone	43.05
	18.351	6.39	Phenol, 2-methyl-5-(l -methylethyl)-	135.1
	20.322	2.96	Copaene	105.1
	21.241	0.23	1H-Cycloprop[e]azulene, 1a,2,3,4,4a,5,6,7b-octahydro-1,1,4,7-tetramethyl-, [1aR-(1aα,4α,4aβ,7bα)]-	105.1
	21.524	2.72	Aromandendrene	41.05
	22.438	4.28	Humulene	93.1
	23.182	16.13	1,6-Cyclodecadiene, 1-methyl-5-methylene-8-(1-methylethyl)-, [S-(E,E)]-	161.15
	23.305	0.25	Naphthalene, 1,2,3,4,4a,5,6,8a-octahydro-4a,8-dimethyl-2-(1-methylethenyl)-, [2R-(2α,4aα,8aβ)]-	41
	23.553	1.75	Gamma-Elemene	121.15
	24.031	0.89	Naphthalene, 1,2,3,5,6,8a-hexahydro-4,7-dimethyl-1-(1-methylethyl)-, (1S-cis)-	161.2
	24.217	5.36	Naphthalene, 1 ,2,3,5,6,8a-hexahydro-4,7-dimethyl-l-(1-methylethyl)-, (1 S-cis)-	119.1
	25.55	1.23	1,6-Cyclodecadiene, 1-methyl-5-methylene-8-(1-methylethyl)-, [S-(E,E)]-	81.1
	25.615	0.62	1H-Cycloprop[e]azulen-7-ol, decahydro-1,1,7-trimethyl-4-methylene-, [1ar-(1aα,4aα,7β,7aβ,7bα)]-	43
	25.751	0.6	Caryophyllene Oxide	41
	26.82	0.59	Geranyl-α-terpinene	119.1
	27.156	0.39	Tau-Cadinol	43
	27.255	1.32	(S,1Z,6Z)-8-Isopropyl-1-methyl-5-methylenecyclodeca-1,6-diene	105.1
	27.464	0.23	τ-Cadinol	43.05
	28.365	2.84	1H-Cycloprop[e]azulene, decahydro-1,1,7-trimethyl-4-methylene-, [1ar-(1aα,4aα,7β,7aβ,7bα)]-	41.05
	28.89	9.16	beta-Neoclovene	161.15
	30.439	1.24	Cis-Calamenene	159.15
	30.654	2.56	Aristol-1(10)-en-9-yl isovalerate	159.15
	33.14	0.27	Aristol-1(10)-en-9-yl isovalerate	43

### Cytotoxicity test (MTT assay)

**Fig. 3:**
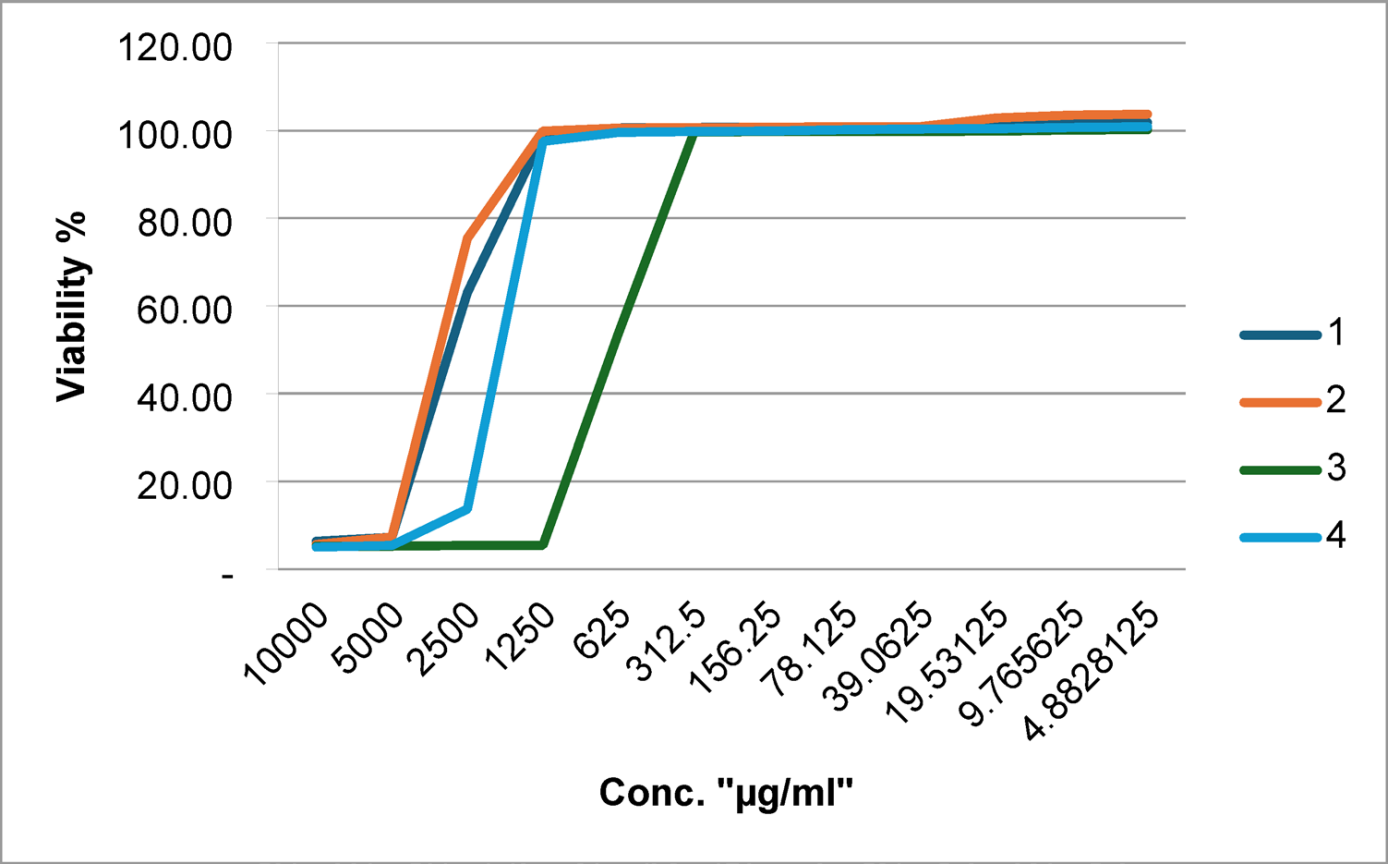
Cytotoxicity of (1) *Mentha canadensis*, (2) *Corymbia citriodora*, (3) *Plectranthus amboinicus* essential oils and (4) their combination on Vero cells, as determined using MTT assay. Vero cells (ATCC, CCL-81) were cultivated in DMEM supplemented with 10% FBS and antibiotics at 37 °C in a 5% CO₂ atmosphere. Cells (1 × 10^4^/well) in 96-well plates were subjected to treatment with 4.88–10,000 µg/mL of *M. canadensis*, *C. citriodora*, *P. amboinicus* essential oils, or their combination for a duration of 24 h. MTT reagent (5 mg/mL) was administered for 4 h, formazan was dissolved in DMSO, and absorbance was measured at 570 nm. Cell viability was assessed in comparison to untreated controls. (n = 3 replicates; plotted values represent mean ± standard deviation (SD)).

The cytotoxicity of *Mentha canadensis*, *Corymbia citriodora*, and *Plectranthus amboinicus* essential oils and their combination was evaluated on Vero cells through the MTT assay. The results demonstrated that the essential oils generally exhibited low cytotoxicity across a broad range of concentrations (4.88 to 10,000 µg/mL). As shown in Fig. 3, the cell viability percentages for all essential oils were above 99% at concentrations 312 µg/ml and below, indicating negligible cytotoxicity at these concentrations. *Mentha canadensis* and *corymbia citriodora* essential oils demonstrated minimal cytotoxicity, with viability percentages above 98% at 1,250 µg/ml *Plectranthus amboinicus* essential oils exhibited notable cytotoxicity at concentrations of 625 µg/ml and above, with cell viability dropping to 53.78%. Notably, the combination of oils maintained high cell viability above 97.55% at 1,250 µg/ml and higher at lower concentrations, suggesting a protective or synergistic effect that reduced toxicity.

### Antiviral activity (cytopathic effect inhibition assay)

**Fig. 4:**
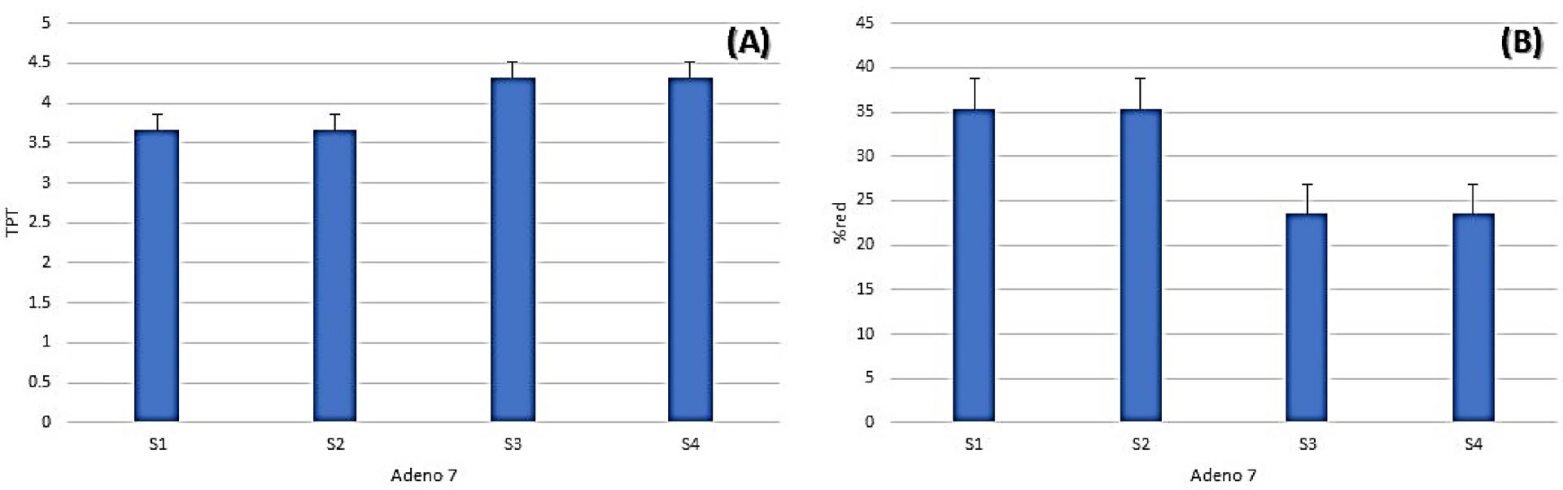
Antiviral activity of (1) *Mentha canadensis*, (2) *Corymbia citriodora*, (3) *Plectranthus amboinicus* essential oils and their combination (4) against Adeno7 virus, evaluated using cytopathic inhibition assay, with oil concentration of 625 µg/ml. (A: TPT value (titer post treatment), B: %red value (percentage of reduction). (n = 3 replicates, plotted values represent mean ± standard deviation (SD)).

The antiviral potential of the essential oils was assessed against Adeno 7 virus via cytopathic effect (CPE) assay. Reduction in viral titer (log10) and percentage of reduction were calculated post-treatment (TPT) compared to untreated controls (Table 2, Fig. 4). *Mentha canadensis* and *corymbia citriodora* essential oils demonstrated inhibition of the Adeno 7 virus with a 35.34% reduction, whereas *Plectranthus amboinicus* essential oil and the combination of the oils showed comparatively lower inhibition at 23.5%.

**Table 2:** Antiviral activity of the essential oils against Adeno 7 virus, highlighting the initial viral titers, titer post treatment (TPT), log differences, and percentage reductions.

Sample	Titer log (10)	TPT	Log Diff	% Reduction
Adeno7 (control)	5.66	–	–	–
*Mentha canadensis* EOs	5.66	3.66	2	35.34
*Corymbia citriodora* EOs	5.66	3.66	2	35.34
*Plectranthus amboinicus* EOs	5.66	4.33	1.33	23.5
EOs combination	5.66	4.33	1.33	23.5

### Evaluation of anti-cancer activity against H1299 cell line

**Fig. 5:**
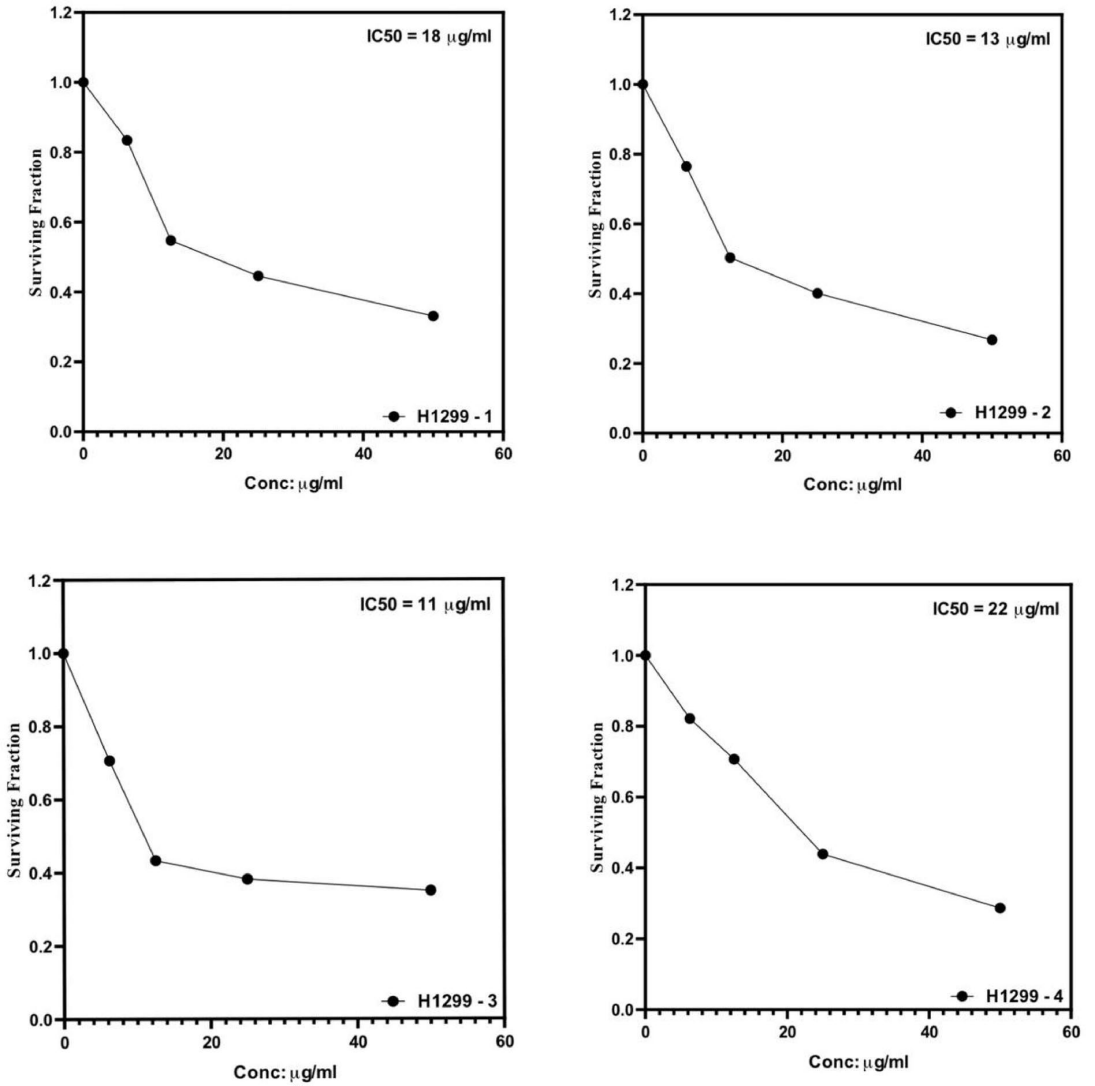
H1299 human non-small cell lung cancer cells were grown in RPMI-1640 media supplemented with 10% FBS and antibiotics at 37 °C and 5% CO_2_. Cells (5 × 10^3^/well) were placed in 96-well plates, let to grow for 24 h, and then treated with a series of dilutions of *M. canadensis*, *C. citriodora* and *P. amboinicus*, essential oils, as well as a mix of the three for 72 h. The control (100% vitality) was cells that had not been treated. The SRB assay was used to measure cytotoxicity, and dose–response curves were used to find IC₅₀ values. (n = 3 replicates).

The anti-cancer activity of essential oils and their combination was evaluated against the H1299 cell line using the Sulforhodamine B (SRB) assay. The cytotoxicity was assessed by determining the half-maximal inhibitory concentration (IC50) values for each sample. The dose–response curves for the tested samples are presented in Fig. 3. The results indicate that *Plectranthus amboinicus* essential oil exhibited the highest cytotoxic activity against H1299 cells with the lowest IC50 value of 11 µg/ml, followed by *Corymbia citriodora* (IC50 = 13 µg/ml) and *Mentha canadensis* (IC50 = 18 µg/ml). However, the combination of all essential oils showed a reduced cytotoxic effect (IC50 = 22 µg/ml) compared to the individual essential oils, suggesting a potential antagonistic interaction when the oils are combined. These findings suggest that the individual oils, particularly *Plectranthus amboinicus* essential oils, demonstrate promising anti-cancer activity against H1299 cells (Fig. 5 – Table 3).

**Table 3:** Cytotoxicity of tested essential oils against H1299 cell line using the Sulforhodamine B (SRB) assay.

Sample	IC50 (µg/mL)
*Mentha canadensis* EOs	18
*Corymbia citriodora* EOs	13
*Plectranthus amboinicus* EOs	11
EOs Combination	22

### Anti-diabetic activity (α-glucosidase inhibition assay)

**Fig. 6:**
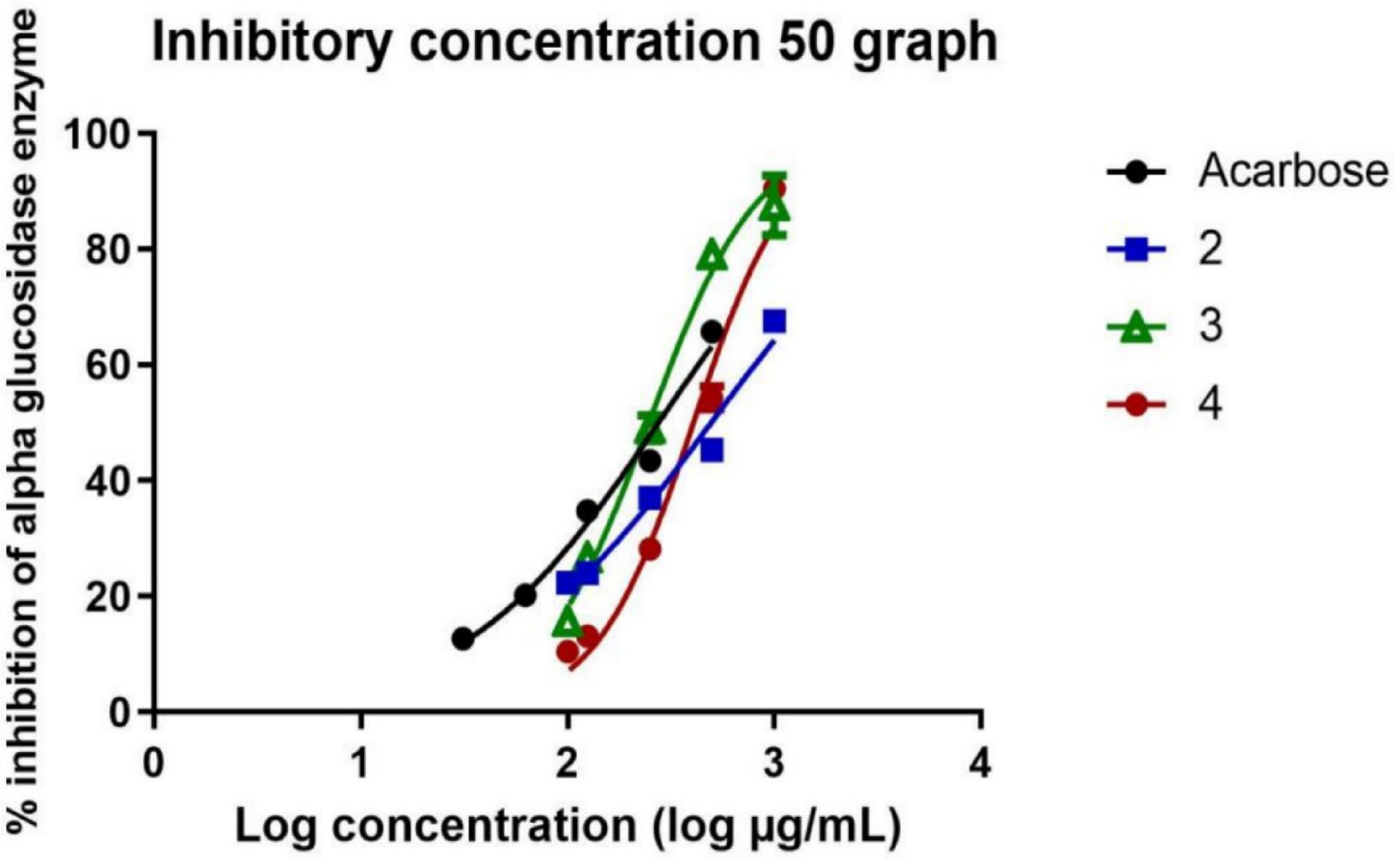
Inhibition of alpha glucosidase enzyme by (2) *Corymbia citriodora*, (3) *Plectranthus amboinicus* essential oils and their combination (4), where the concentration-dependent inhibition by the oils is compared against acarbose. (n = 3 replicates).

The anti-diabetic activity of the essential oils was evaluated by measuring their ability to inhibit α-glucosidase enzyme activity, with acarbose serving as the standard control. The results demonstrated a concentration-dependent inhibition of α-glucosidase activity (Fig. 6, Table 4). *Mentha canadensis* essential oils exhibited negligible inhibitory activity even at the highest tested concentration (1000 µg/mL), indicating its lack of effectiveness in inhibiting α-glucosidase. *Plectranthus amboinicus* essential oils showed the highest inhibition among the individual oils, with an IC50 value of 248.1 ± 1.044 µg/mL., the oils combination also demonstrated notable inhibitory activity with an IC50 value of 407.2 ± 1.043 µg/mL), *Corymbia citriodora* essential oils exhibited moderate activity (IC50 = 496.2 ± 1.061 µg/mL) (Fig. 6, Table 4).

**Table 4:** Inhibitory concentration (IC50) values of tested essential oils and Acarbose against α-glucosidase enzyme.

Sample	IC50 ± SE (µg/mL)
*Corymbia citriodora* EOs	496.2 ± 1.061
*Plectranthus amboinicus* EOs	248.1 ± 1.044
EOs combination	407.2 ± 1.043
Acarbose	219.6 ± 1.030

Acarbose was used as the standard reference inhibitor in line with previously reported α-glucosidase inhibition assay protocols. Its concentration range (10–100 µg/mL) was chosen because it is very strong and can be measured within the linear response range of the assay (as it reaches complete inhibition at relatively low concentrations). Testing acarbose at the same high levels as the essential oils (up to 1000 µg/mL, for example) would completely stop the enzyme from working and make it impossible to determine the IC₅₀.

### Antioxidant activity (DPPH free radical scavenging activity)

**Fig. 7:**
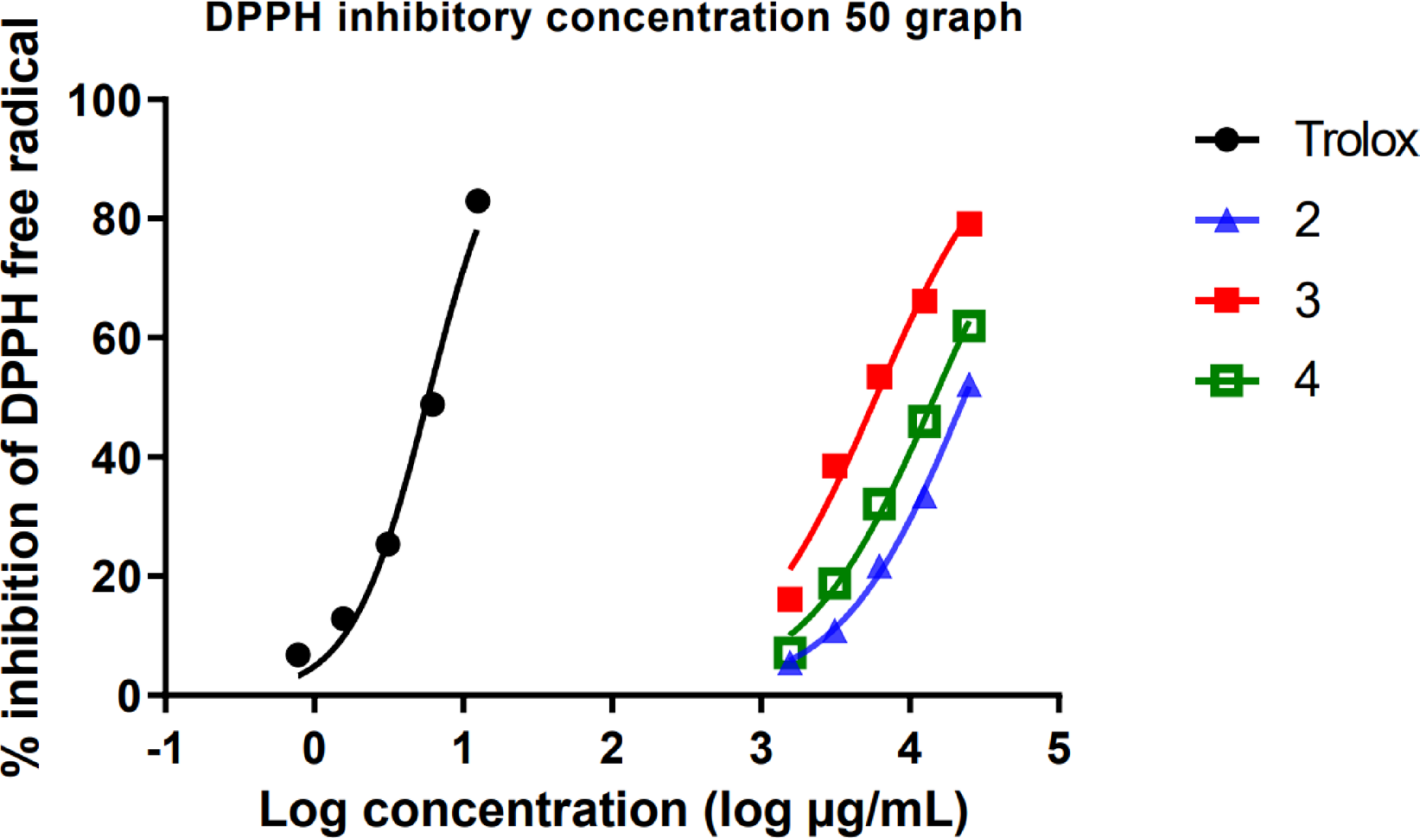
Radical scavenging activity of (2) *Corymbia citriodora*, (3) *Plectranthus amboinicus* essential oils and their combination (4) against DPPH radical (%). (n = 3 replicates).

The antioxidant activity of the essential oils and their combination was evaluated using the DPPH radical scavenging assay. The IC50 values and standard errors are presented in Table 5, and the dose–response curves are shown in Fig. 7. *Mentha canadensis* EOs exhibited only 19.580% inhibition at the highest tested concentration (25 mg/mL), which was insufficient to establish an IC50 value. Its antioxidant capacity was instead expressed as Trolox Equivalent (TE), calculated to be 0.097 ± 0.009 μg TE/mg sample. *Plectranthus amboinicus* EOs demonstrated the highest antioxidant activity with an IC50 of 5923 ± 1.045 μg/mL, followed by the EOs combination (14,638 ± 1.029 μg/mL). The results indicate that *Plectranthus amboinicus* EOs possess the most potent antioxidant activity, which may be attributed to its higher concentration of phenolic or other antioxidant compounds (Fig. 7, Table 5). All these phenolic compounds were mentioned and illustrated in Table 1.

**Table 5:** Radical scavenging activity of tested essential oils against DPPH radical.

Sample	IC50 ± SE (µg/mL)
Trolox	5.822 ± 1.035
*Mentha canadensis* EOs	Not determined (0.097 ± 0.009 µg TE/mg)
*Corymbia citriodora* EOs	23,310 ± 1.019
*Plectranthus amboinicus* EOs	5923 ± 1.045
EOs Combination	14,638 ± 1.029

## Discussion

The principal bioactive components found in the essential oils exhibit established pharmacological properties that may account for the noted antiviral, anticancer, antioxidant, and antidiabetic actions. Menthol, mostly found in *Mentha canadensis*, has been documented to compromise microbial membranes, regulate calcium channels, and provide anti-inflammatory effects by inhibiting the NF-κB pathway^21^. Cyclohexanone derivatives exhibit membrane-permeabilizing characteristics and may impede bacterial proliferation by destabilizing lipid bilayer integrity^22^. Thymol, present in *Plectranthus amboinicus*, is a phenolic monoterpene that compromises cell membrane integrity, facilitates the leaking of intracellular components, and triggers death in cancer cells by mitochondrial depolarization and reactive oxygen species (ROS) formation^23^. Citronellal and 6-octenal (found in large amounts in *Corymbia citriodora*) are very effective in killing bacteria by breaking down membranes and causing oxidative stress. They have also been linked to anti-inflammatory and insect-repellent qualities^24,25^. Esters, including propanoic acid and 2-methyl-butyl ester, may function as enzyme inhibitors, thereby facilitating α-glucosidase inhibition and exhibiting antidiabetic properties^26^. The existence of these substances together shows that they can work in several ways, such as destabilizing membranes, changing oxidative stress, and inhibiting enzymes. These effects may work together or add up in the bioactivities that were examined.

### GC–MS bioassay

The GC–MS analysis showed that the essential oils of *M. canadensis*, *P. amboinicus*, and *C. citriodora* have diverse chemical profiles. This could explain why they have varied biological effects. The oil from *M. canadensis* has high quantities of levomenthol (10.21%) and cyclohexanone derivatives (13.24–16.66%), which are known to break down bacterial cell membranes and make them more permeable, which can cause the contents of the cells to leak^22^. Thymol (6.39%) and esters such as propanoic acid and 2-methyl-butyl ester, as well as monoterpenoids like 1,6-octadien-3-ol and 3,7-dimethyl, are said to damage bacterial membranes and may stop enzymes from working, which slows down microbial metabolism^26,27^. *C. citriodora* oil, on the other hand, was mostly made up of 6-octenal (84.06%) and citronellol (9.0%). Both of these compounds have been shown to be quite effective against bacteria, probably by getting into the lipid bilayer and changing the membrane potential^25^. Cyclohexanol and 5-methyl-2-(1-methylethyl)-, found in all samples, are particularly noteworthy since they have strong antibacterial properties. This makes it more likely that the different parts of the oil work together to make them stronger. These results corroborate the concept that the antibacterial properties of these essential oils are predominantly facilitated by molecules that target microbial membranes and critical enzyme functions.

### Antiviral activity

With a 35.34% decrease in viral titer, the essential oils (EOs) of *Corymbia citriodora* and *Mentha canadensis* demonstrated modest antiviral efficacy against the Adeno 7 virus. As been previously noted with terpenoid-rich essential oils, the antiviral effect may result from the rupture of the viral envelope or interference with viral replication^28^. Interestingly, the combination and *Plectranthus amboinicus* EO demonstrated reduced inhibition (23.5%), which may have been caused by antagonistic interactions or lower quantities of active antiviral components such thymol and carvacrol^29^. Because citronellal, the main constituent, has been demonstrated to suppress respiratory viruses by disrupting their viral envelope, recent studies have supported the antiviral activity of *C. citriodora* oil^30^. Likewise, *P. amboinicus* contains thymol and eugenol, which have mild antiviral properties but could need greater concentrations to work^31^.

### Cytotoxicity (MTT assay)

At lower concentrations (≤ 312 µg/mL), the essential oils were generally non-toxic to Vero cells, which is advantageous for therapeutic applications. But at ≥ 625 µg/mL, *P. amboinicus* EO demonstrated significant cytotoxicity, lowering cell viability to 53.78%. This is consistent with research showing that at greater concentrations, EOs rich in thymol can damage mammalian membranes^32^. At greater dosages, the oils of *Plectranthus amboinicus* maintained high cell viability (> 97.55%), indicating a protective action that works in concert. These kinds of interactions can lessen the effects of specific cytotoxic components or alter membrane permeability^33^.

### Anticancer activity

All tested EOs displayed cytotoxicity against the H1299 lung cancer cell line, with *P. amboinicus* showing the highest potency (IC_50_ = 11 µg/mL). This is consistent with its known bioactive compounds such as thymol and carvacrol, which induce apoptosis in cancer cells via mitochondrial pathways^34^. *M. canadensis*and *C. citriodora* also exhibited notable anticancer activities, but the EOs combination showed reduced potency (IC_50_ = 22 µg/mL), suggesting antagonistic interactions that dampen the efficacy. Similar antagonistic effects among mixed EOs have been reported by Elsharkawy et al.^35^, indicating the need for component-specific synergy assessments in combinations.

### Antidiabetic activity

*Plectranthus amboinicus* EO demonstrated the strongest α-glucosidase inhibition (IC_50_ = 248.1 µg/mL), followed by the combination and *C. citriodora*. *M. canadensis* showed negligible activity. The α-glucosidase inhibitory potential of *P. amboinicus* may be attributed to terpenoids such as citronellol and isopulegol, known to interfere with carbohydrate metabolism^36^. Although less potent than acarbose (IC_50_ = 219.6 µg/mL), the natural origin and lower toxicity of these oils could make them suitable for adjunct therapies in diabetes management^37^.

### Antioxidant activity

At lower concentrations (≤ 312 µg/mL), the essential oils were generally non-toxic to Vero cells, which is advantageous for therapeutic applications. But at ≥ 625 µg/mL, *P. amboinicus* EO demonstrated significant cytotoxicity, lowering cell viability to 53.78%. This is consistent with research showing that at greater concentrations, EOs rich in thymol can damage mammalian membranes^32^. It’s interesting to note that even at greater dosages, the oils maintained high cell viability (> 97.55%), indicating a protective action that works in concert. These kinds of interactions can lessen the effects of specific cytotoxic components or alter membrane permeability^33^.

## Conclusion

This research underscores the considerable therapeutic potential of essential oils extracted from *Mentha canadensis, Corymbia citriodora*, and *Plectranthus amboinicus*. The oils show differing levels of antiviral, antioxidant, antidiabetic, and anticancer properties, illustrating their extensive bioactivity. *Plectranthus amboinicus* demonstrated the most potent anticancer, antioxidant and antidiabetic properties, while *Mentha canadensis* and *Corymbia citriodora* displayed significant antiviral capability. Although individual essential oils exhibited promise efficacy, their combination did not consistently yield increased bioactivity, indicating potential antagonistic interactions. Furthermore, all evaluated oils exhibited acceptable cytotoxicity profiles at therapeutic amounts. These findings endorse the possibility of these plant-derived essential oils as candidates for the formulation of alternative or complementary medicinal treatments. Additional research, encompassing in vivo evaluations and mechanism-oriented studies, is necessary to confirm their therapeutic relevance and safety.

In terms of anticancer, antioxidant and antidiabetic qualities, *P. amboinicus* was shown to be the most physiologically active essential oil overall, *C. citriodora* had mild antidiabetic activity. The intricacy of synergistic vs antagonistic interactions was highlighted by the fact that the combination of EOs occasionally led to decreased bioactivity.

The current work aims to achieve four goals of the sustainable development goals (SDG) as follow: SDG 3: Good Health and Well-being: The study examines the antiviral, antioxidant, anticancer, and antidiabetic activities of essential oils derived from *Mentha canadensis*, *Corymbia citriodora*, and *Plectranthus amboinicus*. This enhances access to cost-effective, efficacious medicines. SDG 12: Responsible Consumption and Production: The study employs in vitro culture and natural product extraction to enhance the sustainable procurement of medicinal plants, hence reducing the ecological impact of pharmaceutical research. This promotes diminished reliance on synthetic pharmaceuticals and cultivates environmentally sustainable methods for health product innovation. SDG 9: Industry, Innovation, and Infrastructure: The study encompasses advanced biochemical screening, GC–MS analysis, and bioactivity assessment, hence facilitating research, innovation, and development within the biomedical and pharmaceutical sectors. This enhances scientific research infrastructure and innovation capabilities, especially in phytopharmacology. SDG 15: Life on Land: It promotes for in vitro propagation and the scientific validation of traditional medicinal plants, thereby facilitating biodiversity conservation and equitable utilization of genetic resources. It underscores the therapeutic significance of indigenous plant species, perhaps mitigating overexploitation in natural habitats.

## Data Availability

No datasets were generated or analysed during the current study.
